# Supplementary material for: Contribution of Different Food Types to Vitamin A Intake in the Chinese Diet
Source: Nutrients. 2023 Sep 17;15(18):4028. doi: 10.3390/nu15184028 (PMC10535670; doi:10.3390/nu15184028)
Supplement: Supplementary file 1 [file nutrients-15-04028-s001.zip › nutrients-2562135-supplementary.pdf]

Contribution of Different Food Types to Vitamin A Intake in the Chinese Diet

Xue Li [1,2], Can Guo [1,2], Yu Zhang [1,2], Li Yu [1,2], Fei Ma [1,2], Xuefang Wang [1,2], Liangxiao Zhang [1,2,3,4,*] and Peiwu Li [1,2,3,4,5]

1 Key Laboratory of Biology and Genetic Improvement of Oil Crops, Ministry of Agriculture and Rural
Affairs, Oil Crops Research Institute, Chinese Academy of Agricultural Sciences, Wuhan 430062, China; 15071198582@163.com (X.L.); guocan2020@163.com (C.G.); 15110662878@163.com (Y.Z.); yuli01@caas.cn (L.Y.); mafei01@caas.cn (F.M.); wangxuefang01@caas.cn (X.W.); peiwuli@oilcrops.cn (P.L.)

2 Quality Inspection and Test Center for Oilseed Products, Ministry of Agriculture and Rural Affairs, Oil Crops Research Institute, Chinese Academy of Agricultural Sciences, Wuhan 430062, China

3 College of Food Science and Engineering, Collaborative Innovation Center for Modern Grain Circulation and Safety, Nanjing University of Finance and Economics, Nanjing 210023, China

4 Hubei Hongshan Laboratory, Wuhan 430070, China

5 Xianghu Laboratory, Hangzhou 311231, China

* Correspondence: zhanglx@caas.cn; Tel.: +86-27-86812862; Fax: +86-27-86812862

Supplementary materials

Table S1 Vitamin A content of main foods

Table S2 The amount of vegetables for recommended nutrient intake of vitamin A by replacing all vegetables

Table S3 The amount of vegetables for recommended nutrient intake of vitamin A

Table S1 Vitamin A content of main foods

Food Categories	Foods	Vitamin A content (ugRAE)/100g
Coarse cereals	Soba flour	2
	Oat flour	2
Potato	Potato	1
	Sweet potato	18
Bean	Soybean	18
	Mung bean	11
	Red bean	7
Vegetable	Carrot	342
	Kidney bean	18
	Cowpea	10
	Eggplant	4
	Tomato	31
	Red pepper	116
	Green pepper	8
	Sweet pepper	6
	Cucumber	8
	Pumpkin	74
	Chinese chives	133
	Cabbage	6
	Broccoli	13
	Spinach	243
	Asparagus lettuce	13
	Lettuce	63
Fruits	Apple	4
	Banana	3
	Pear	2
	Grape	3
	Peach	2

	Tangerine	15
	Orange	13
	Grapefruit	24
	Cherry	18
Meats	Beef	3
	Chicken	92
	Pork	15
	Mutton	8
Eggs	Egg	255
	Duck egg	261
	Goose egg	192
	Quail egg	337
Milk	Milk	54
	Whole milk powder	380
Aquatic Products	Fish	53
	Shrimp and Crab diver	488
	Shellfish	50
	Algae	-

Table S2 The amount of vegetables for recommended nutrient intake of vitamin A by replacing all vegetables

Vegetables	Vitamin A content (ugRAE)-100g	The amount of vegetables for women (g)	The amount of vegetables for men (g)
Carrot	342	144	173
Kidney bean	18	2736	3292
Cowpea	10	4925	5925
Eggplant	4	12312	14812
Tomato	31	1589	1911
Red pepper	116	425	511
Green pepper	8	6156	7406
Sweet pepper	6	8208	9875
Cucumber	8	6156	7406
Pumpkin	74	666	936
Chinese chives	133	370	445
Cabbage	6	8208	9875
Broccoli	13	3788	4557
Spinach	243	203	244
Asparagus lettuce	13	3788	4577
Lettuce	63	782	940

Table S3 The amount of vegetables for recommended nutrient intake of vitamin A

Vegetables	Vitamin A content (ugRAE)-100g	Recommended vitamin A intake (ugRAE) for women/men	The amount of vegetables for women/ men (g)
Carrot	342	342.92/442.92	100/130
Kidney bean	18	240.78/340.78	1338/1893
Cowpea	10	239.51/339.51	2395/3395
Eggplant	4	240.67/340.67	6017/8517
Tomato	31	251.50/351.50	811/1134
Red pepper	116		
Green pepper	8	258.44/358.44	199/276
Sweet pepper	6		
Cucumber	8	242.53/342.53	3031/4282
Pumpkin	74	248.47/348.47	336/471
Chinese chives	133	241.45/341.45	182/257
Cabbage	6	240.82/340.82	4014/5680
Broccoli	13	241.14/341.14	1855/2624
Spinach	243	306.27/406.27	126/167
Asparagus lettuce	13	251.27/351.27	331/462
Lettuce	63		
